# Role of Anterior Cingulate Cortex in Instrumental Learning: Blockade of Dopamine D1 Receptors Suppresses Overt but Not Covert Learning

**DOI:** 10.3389/fnbeh.2017.00082

**Published:** 2017-05-15

**Authors:** Mayada Aly-Mahmoud, Pascal Carlier, Sherine A. Salam, Mariam Houari Selmani, Marie Z. Moftah, Monique Esclapez, Driss Boussaoud

**Affiliations:** ^1^Aix Marseille Univ, INSERM, INS, Inst Neurosci SystMarseille, France; ^2^Department of Zoology, Faculty of Science, Alexandria UniversityAlexandria, Egypt; ^3^Clinical Neurosciences Laboratory, Faculty of Medicine and Pharmacy, University Sidi Mohamed Ben AbdellahFez, Morocco

**Keywords:** associative learning, medial prefrontal cortex, effort tolerance, rodent, observational learning

## Abstract

**HIGHLIGHTS**

- Blockade of dopamine D1 receptors in ACC suppressed instrumental learning when overt responding was required.
- Covert learning through observation was not impaired.
- After treatment with a dopamine antagonist, instrumental learning recovered but not the rat's pretreatment level of effort tolerance.
- ACC dopamine is not necessary for acquisition of task-relevant cues during learning, but regulates energy expenditure and effort based decision.

Dopamine activity in anterior cingulate cortex (ACC) is essential for various aspects of instrumental behavior, including learning and effort based decision making. To dissociate learning from physical effort, we studied both observational (covert) learning, and trial-and-error (overt) learning. If ACC dopamine activity is required for task acquisition, its blockade should impair both overt and covert learning. If dopamine is not required for task acquisition, but solely for regulating the willingness to expend effort for reward, i.e., effort tolerance, blockade should impair overt learning but spare covert learning. Rats learned to push a lever for food rewards either with or without prior observation of an expert conspecific performing the same task. Before daily testing sessions, the rats received bilateral ACC microinfusions of SCH23390, a dopamine D1 receptor antagonist, or saline-control infusions. We found that dopamine blockade suppressed overt responding selectively, leaving covert task acquisition through observational learning intact. In subsequent testing sessions without dopamine blockade, rats recovered their overt-learning capacity but not their pre-treatment level of effort tolerance. These results suggest that ACC dopamine is not required for the acquisition of conditioned behaviors and that apparent learning impairments could instead reflect a reduced level of willingness to expend effort due to cortical dopamine blockade.

## Introduction

Learning the contingencies between behavior and environmental events through associative learning is a fundamental adaptive capacity that allows animals to predict outcomes of stimuli and actions through conditioning. The prefrontal cortex and basal ganglia play a central role in the neuronal processes of associative learning, but the relative contributions of the components of this complex network remain unclear. This uncertainty is exemplified by the debates on the role of anterior cingulate cortex (ACC), a medial prefrontal region that has been involved in a variety of cognitive tasks, including learning. Lesions of ACC in rats (Bussey et al., 1996; Parkinson et al., 2000) and monkeys (Rushworth et al., 2003; Walton et al., 2003), or pharmacological manipulations of its activity (Schweimer and Hauber, 2006; McKee et al., 2010), have led to the conclusion that ACC is required for learning instrumental tasks, but is less necessary for their performance once they have been learned. This idea has led to the widely shared view that ACC is used to acquire new behaviors through the processing of errors and rewards (Gabriel et al., 1991; Gehring et al., 1993; Coles et al., 1998; Cardinal et al., 2003), which receives support from lesion studies (Kennerley et al., 2006) as well as neurophysiological findings in monkeys showing that ACC neurons are involved in reward encoding and outcome monitoring during learning (Amiez et al., 2006; Quilodran et al., 2008; Hayden et al., 2011; Kawai et al., 2015). However, the role of ACC in associative learning is still a matter of controversy. For example, Jonkman and Everitt (2009) found that post-session blockade of ACC plasticity with microinfusions of anisomycin, a protein-synthesis inhibitor, did not impair instrumental learning. By contrast, McKee et al. (2010) found that intra-ACC blockade of NMDA receptors prevented instrumental learning. Although, the two studies used different pharmacological manipulations, they nevertheless reached opposite conclusions as to whether ACC plays a role during the acquisition of action-outcome associations. On the one hand, Jonkman and Everitt (2009) suggested that ACC is not necessary for learning, whereas, on the other hand, McKee et al. (2010) concluded that ACC is required for learning. Furthermore, ACC's involvement in learning may also depend on the task used. For example, Ragozzino and Rozman (2007) found that inactivation of ACC does not impair stimulus-reward association learning, but instead impairs reversal learning selectively. Accordingly, there is an ongoing debate about whether ACC is involved in learning and, if so, what type of learning. The prevailing view is that ACC is required for action-reward associations, whereas other prefrontal regions, namely the orbitofrontal cortex (OFC), is thought to play a more prominent role in learning stimulus-response associations (see Rushworth et al., 2007; Bissonette and Roesch, 2015). In addition, considerable evidence implicates ACC in the regulation of effort needed to gain rewards (Cousins and Salamone, 1994; Walton et al., 2003; Floresco et al., 2008). In particular, studies using a T-maze cost–benefit task have shown that contrary to control rats which choose more often the high cost—high reward option, ACC-lesioned rats more often select the low cost—low reward option (Walton et al., 2003; Schweimer et al., 2005).

Both learning and effort-based functions are dependent on dopamine activity. In monkeys, prefrontal cortex dopamine activity mediates associative learning (Puig and Miller, 2012, 2014), working memory (Williams and Goldman-Rakic, 1995), and attention (Noudoost and Moore, 2011). In rodents, several studies have suggested that dopamine plays a key role in regulating the willingness to expend effort for food reward (Salamone et al., 2007; Salamone and Correa, 2012; Berridge and O'Doherty, 2013). In particular, blockade of ACC dopamine D1 receptors (D1Rs) impairs the willingness of rats to expend effort for high reward, as evaluated by the T-maze cost–benefit task (Schweimer and Hauber, 2006). In short, D1Rs in ACC seem to regulate effort tolerance, which we will use to refer to the willingness to expend effort.

Observational learning (Heyes and Dawson, 1990; Brosnan and de Waal, 2004; Subiaul et al., 2004; Meunier et al., 2007; Monfardini et al., 2008, 2012, 2013; Bellebaum et al., 2010, 2012; Burke et al., 2010; Isbaine et al., 2015), which is mediated by the same dopamine-dependent mechanisms involved in reinforcement learning (Holroyd and Coles, 2002; Holroyd et al., 2004; Nieuwenhuis et al., 2004; Holroyd and Yeung, 2011; Walsh and Anderson, 2012), provides an opportunity to examine the role of ACC dopamine in learning *per se*, in isolation from the regulation of effort tolerance. Accordingly, here we contrasted instrumental, trial-and-error learning (TE), which requires both physical and cognitive effort, with observational learning (LeO). The concept of *overt learning* applies to both TE and the execution phase of LeO; *covert learning* applies to the observation phase of LeO.

## Materials and methods

### Subjects and experimental groups

Thirty male Long—Evans rats from Charles River Laboratories were used in this study. Their weight was 250–300 g at the beginning of the experiment. They were pair-housed in clear plastic cages measuring 26 × 18 × 22 cm, with wire mesh at the top through which they had access to water and food pellets. The cages were lined by approximately 3.0 cm in depth sterile wood chips bedding manufactured by LIGNOCEL®, which was changed once a week. The cages were placed on shelving unit in a room within the animal facility of the Fédération de Recherche 3C (CNRS & Aix Marseille University, FR 3512). The room was maintained at a temperature of 22 ± 1°C and a humidity rate of 20–40%, with a 12 h light-dark cycle (light on at 7:00 AM). The rats were food restricted to maintain their weight at approximately 85% of their *ad libitum* weight but had free access to water throughout the experiment. They were tested during the light-on period, in a sound and light attenuated room, separate from the housing room. The experiments were conducted according to the guidelines of the EU Directive 2010 on animal experimentation, and they were approved by the local ethics committee (authorization # 01294.03).

Twenty-four rats learned an instrumental action-reinforcer task, where lever-pushes in one out of two directions were rewarded with tasty food (pieces of biscuit). Twelve animals learned through trial-and-error (TE), whereas 12 learned through observation (LeO). TE and LeO rats were divided into 2 groups: control groups (TE-C, LeO-C) received bilateral microinfusions of Saline in ACC (0.25 μl per side); experimental groups (TE-SCH, LeO-SCH) were infused with dopamine D1 receptors antagonist SCH23390 hydrochloride (Abcam®, Cambridge, UK). Six expert rats were used as demonstrator for the LeO rats.

### Apparatus and testing procedure

**Figure 1:**
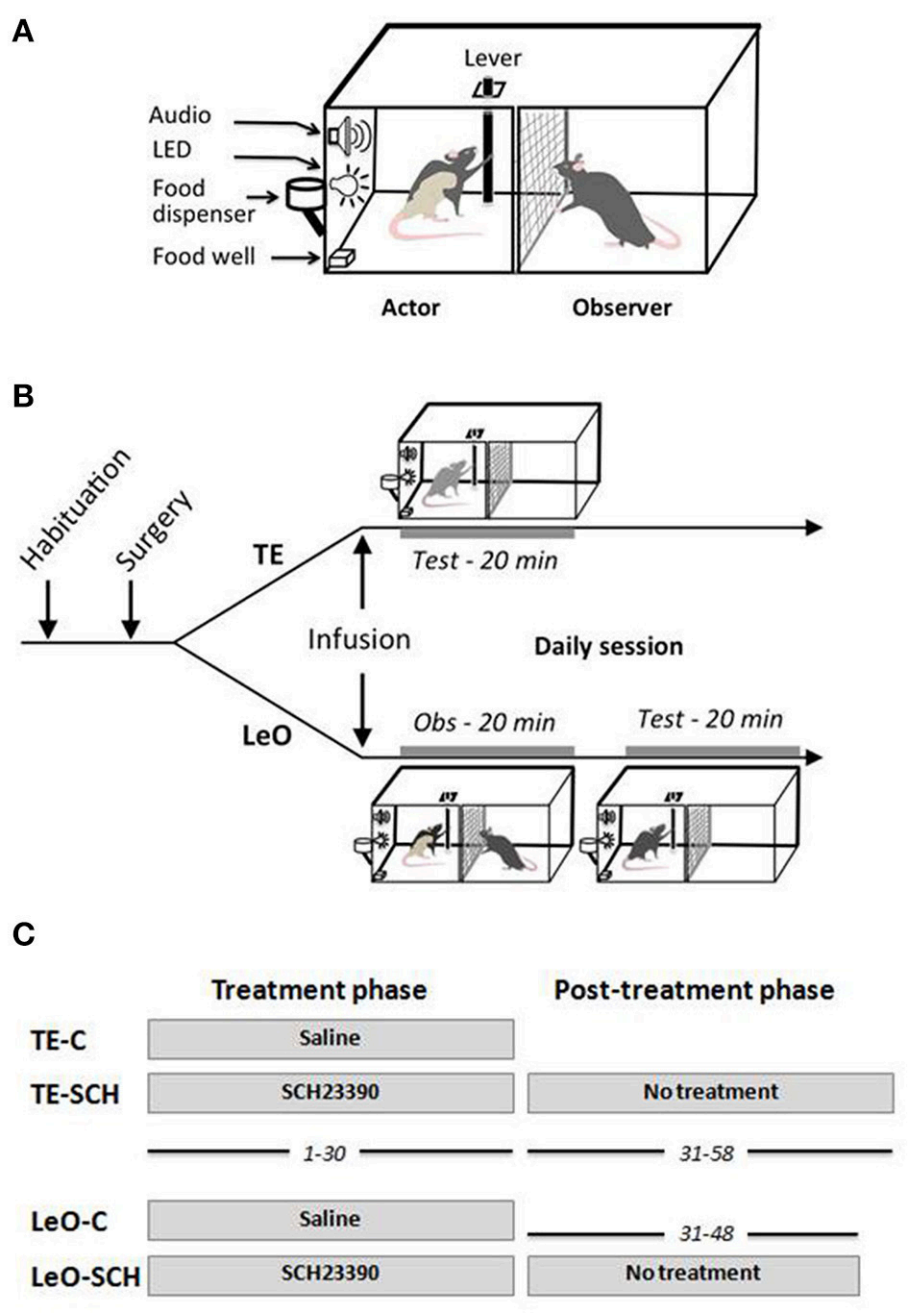
**Testing apparatus and behavioral procedures. (A)** *Testing apparatus*. **(B)** *Behavioral procedure: daily session*. After habituation and surgical implantation of the cannulas in ACC, rats received bilateral microinfusions of saline or SCH23390 before each testing session. Immediately after microinfusions, the rats were tested directly (TE, trial-and-error) for 20 min, or put in the observer compartment (Obs) for observation of a demonstrator for 20 min (LeO, learning by observation), then tested in the actor compartment. **(C)** *Treatment and post-treatment testing*. All rats received microinfusions before testing for 30 days of testing, during sessions 1–30. From session 31–58, rats in the experimental groups, LeO-SCH and TE-SCH, were tested for an additional 18 and 28 days, respectively, but without any treatment.

The rats were tested in a homemade apparatus (see Figure 1) inspired by Heyes and Dawson (1990). It consisted of two identical compartments measuring 30 × 30 × 30 cm, separated by a wire mesh, which allowed the rats to communicate through sight, touch, smell and hearing. One compartment was designed for the “actor” rat, the other for the “observer.” The front wall of the two compartments was made from clear Perspex allowing the experimenter to watch the whole test and to videotape the rats during testing. The “observer” compartment did not contain any equipment, whereas the “actor” compartment contained a suspended lever, a food well, 2 LEDs and a loud speaker. The lever can be pushed only in two directions along the wall separating the two compartments: thus, from the observer's view, the lever can be displaced either left (toward the front door), or right. When the lever was pushed at least 5 cm in the correct direction (right), it triggered a low frequency beep together with a green LED signaling the correct action, and indicating that a reward was delivered in the food well. If the lever was pushed in the incorrect direction (left), it triggered two error signals, a high frequency beep and onset of a red LED. The testing apparatus was equipped with a trial counter which scored automatically correct and incorrect lever pushes (LPs). All the demonstrators were trained to be “right pushers,” and thus the observers were expected to learn to push the lever to the right for food reward.

### Training of the demonstrators

Six demonstrators, like all the rats involved in the study, were first habituated to the testing apparatus, and then trained (see Supplementary Methods) until they reached a stable performance of at least 90% correct LPs in individual sessions, with an average of 3.2 LPs per min. They were all trained to lever-push to the right, and were used in a pseudorandom schedule with the different learners in order to keep familiarity at a low level.

### Surgery

#### Implantation of the cannulas in ACC

**Figure 2:**
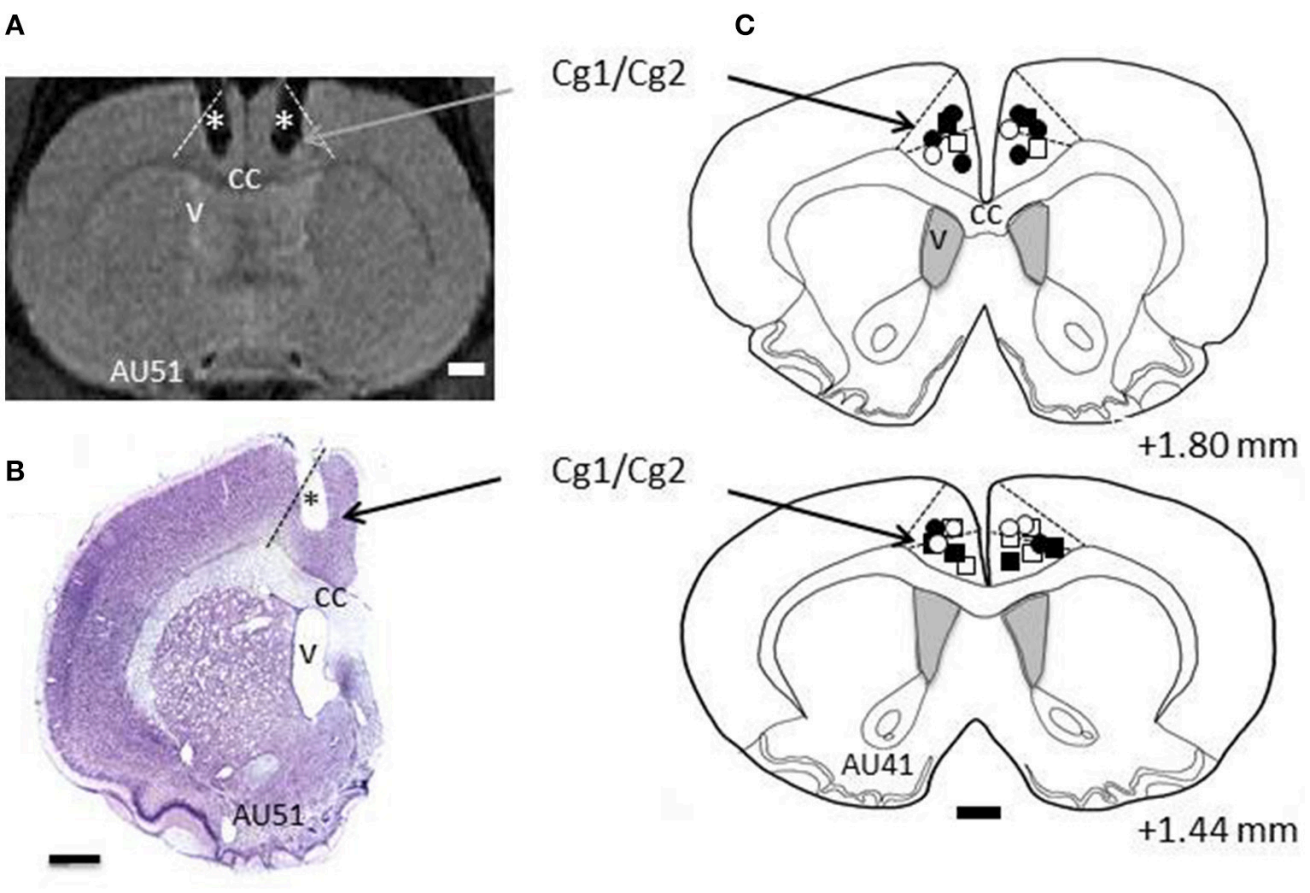
**Histology. (A)** Example section taken from Magnetic Resonance Imaging (MRI) scans. L and R refer to left and right hemispheres, respectively. The dashed lines indicate the border of ACC (Cg1/Cg2). The stars depict the trace of the cannulas as revealed by MRI. **(B)** Example of a cresyl violet stained section from rat AU51's brain showing the trace of the cannula (^*^). Dashed line indicates the border of ACC. **(C)** Reconstructions of the tips of the cannulas for all the rats included in this study, shown on two coronal sections taken from rat AU41. The numbers indicate the AP levels relative to bregma. Squares and circles indicate injection sites for TE and LeO rats, respectively; Open and filled (black) symbols are used for saline and SCH23390 injections, respectively. cc, corpus callosum; v, ventricle.

All rats, except the demonstrators, underwent surgery for implantation of 2 cannulas, bilaterally in ACC. Surgery was performed under deep anesthesia using a mixture of Xylazine (rompun 2%; Bayer, Colombia) and ketamine (50 mg/ml, Merial, France), in a 1:1 proportion. During surgery, the rat was placed in a stereotaxic apparatus, and body temperature was maintained at 37°C using a heating pad. Holes of less than 1 mm diameter were drilled in the skull bilaterally at +1.56 anterior to the bregma, ±0.8 mm lateral to midline. Twenty-two gauge, 2 mm long cannulas were inserted through the holes using the stereotaxic apparatus, with an angle of 10° to the vertical axis. According to the Atlas of Paxinos and Watson (2007), the tip of the cannula was placed at the Cg1/Cg2 border. The cannulas were sealed to the skull using dental cement and bone screws, together with a 2 mm screw head inside the cement, used with a head holder during the microinfusions. Each cannula was secured with a cap equipped with a dummy guide extending inside the cannula. The cannulas were made in stainless steel material, or in plastic MRI compatible material. Fifteen rats were implanted with plastic cannulas, whose locations were checked immediately at the end of implantation using the MRI scanner in the animal facility. For the other rats, confirmation came after histological processing of brain sections (see Figure 2). After surgery, the rats were allowed 1-week recovery time during which they received antibiotic and analgesic treatment.

#### Microinfusion procedure

After post-surgical recovery, rats were tested on a daily basis, following the procedure summarized in Figure 1. Depending on the group, the animals received either Saline or SCH23390 microinfusions bilaterally just before testing. Each rat was gently constrained using a rod fixed on the screw implanted on the skull. The caps were removed and 28-gauge injectors were inserted in the cannulas, one at a time, with the tip protruded 1.0 mm beyond the cannulas inside the brain tissue. Each injector was attached to a 10.0 μl Hamilton syringe via a PE_20_ polyethylene tubing, connected to a microinfusion pump (New Era Pump Systems, Inc, USA) used to control the infusion rate and volume. A volume of 0.25 μl was infused in each side over 2 min, and the injector was left in place for 1 min after completion of the infusion to allow time for diffusion before withdrawal. LeO and TE groups received D1Rs antagonist SCH23390 hydrochloride, which was dissolved in 0.09% NaCl at a concentration of 1 μg/1 μl. Control animals received the same volume of 0.09% NaCL solution.

### Behavioral task design

Before each daily session (see Figure 1), TE rats were placed directly in the actor's compartment 2 min after microinfusions, and tested for 20 min. LeO rats, by contrast, were first placed in the observer compartment 2 min after infusions for a 20 min observation period during which the demonstrator performs the task (60–80 rewarded trials, average 3.2 trials per min). Then, the demonstrator was removed, the actor's compartment was thoroughly cleaned with a wet towel to remove as much olfactory cues as possible, before introducing the observer rat for a 20 min testing period as for the TE groups. The rat's behavioral performance was scored in the same way for all the groups, and the animals were videotaped for off line examination.

#### Treatment/post-treatment phases, number of sessions

There were two testing phases: the treatment phase, and the post-treatment phase (Figure 1C). The treatment phase lasted 30 sessions, during which the rats received microinfusions of SCH23390 or Saline before daily testing (see above). All animals were tested during this phase. During the post-treatment phase, there were no microinfusions. Here, because control rats (TE-C and LeO-C) learned during the treatment phase, only the experimental rats were tested for an additional number of sessions until they reached learning criterion and stable performance (LeO-SCH, 18 sessions; TE-SCH, 28 sessions).

#### Control experiments for aversive conditioning

At the end of testing, a control experiment was conducted in 4 rats (1 TE-SCH, 1 Saline and 2 LeO-SCH), aiming to test for potential deficits in reward liking, and for aversive condition. Two rats received intra-ACC microinfusions of Saline, the others received SCH23390 infusions bilaterally and following the exact same procedure as described in Methods. After infusions, they were placed in their home cage and given new palatable foods. One new food (chocolate cereal) was paired with Saline infusions; another new food (sweet cereal) was paired with SCH23390 infusions. This experiment was repeated for 3 consecutive days, and on the 4th and 5th days, the rats received the same foods but without infusions.

### Data collection and analysis

Multiple behavioral variables were scored and analyzed using a multiple analysis of variance (MANOVA). They included the number of *lever pushes* (LPs) to the right (rewarded trials) the number of *incorrect LPs* (left pushes that triggered error feedback), the total number of *correct lever contacts*, total number of *incorrect lever contacts*, and the *latency* of the 1st lever contact. We also computed a *percentage of correct responses* (*PCR* = *correct LPs / total LPs)*, to access the progression of learning and to determine the *learning criterion*, defined as *the 1st of 5 consecutive sessions with at least 75% correct LPs*. Finally learning speed was measured as the *number of sessions to criterion*.

The data were analyzed in order to compare the behavioral variables across groups, using a repeated measures 3-way MANOVA with two between subject factors (*treatment*, Saline vs. SCH23390; *learning type*, TE vs. LeO) and one within subject factor (*session*). MANOVAs provided a general evaluation of significant variations in all the dependent variables measured, and were followed by separate univariate ANOVAs to test the significance of individual behavioral variables, especially the numbers of LPs and the latencies. We also used the Tukey HSD *post-hoc* analysis for each dependent variable. In addition, we provide the effect size given by *eta-squared (*η^2^*)*, which measures the variance explained in the dependent variable by a factor (e.g., Treatment) while controlling for other factors (e.g., Learning Type). Finally, for analysis of significance of the variations in the number of sessions to reach learning criterion, as it is not a continuous variable, we used a non-parametric test (Mann-Whitney *U*-test). The significance level was set to a *P* < *0.05*.

### *In vivo* magnetic resonance imaging acquisition and histology

All experiments were performed on a 70/16 pharmascan spectrometer (BRUKER Biospin, Ettlingen, Germany) equipped with a 7-Teslas magnet and 16-cm horizontal bore size. A linear birdcage coil with 62-mm inner diameter was used for signal transmission and reception. Magnetic Resonance Imaging (MRI) scans started immediately after the end of surgical procedure, using a three-dimensional turbo-RARE sequence (TE_eff_ = 46 ms, TR = 2,000 ms, rare factor = 16, 3 averages, fat suppression bandwidth = 900 Hz) with a 35 × 35 × 8 mm^3^ Field Of View and 256 × 256 × 20 matrix. Body temperature was kept at 37 ± 1°C with a heating blanket and a pressure probe monitored the rat's respiration.

After completion of behavioral testing, the rats were deeply anesthetized with chloral hydrate (500 mg/kg; Fisher Scientific) and transcardially perfused with a fixative solution of 4% paraformaldehyde in 0.12M sodium phosphate buffer, pH 7.4 (PB). Following perfusion, the brains were removed from the skull, post-fixed in the same fixative for 1 h at room temperature and rinsed in PB for 1.5 h. Blocks of brains were immersed in a cryoprotective solution of 20% sucrose overnight at 4°C, frozen on dry ice and sectioned coronally at 40 μm with a cryostat. Sections were stained with cresyl violet in order to determine the injection sites as well as the general histological characteristics of the tissue within the rostro-caudal extent of the brain.

For each animal, the injection sites were localized on cresyl violet-stained sections (see example on Figure 2). All sections displaying the traces of the cannulas used for injections were drawn using a computer-assisted system connected to a Nikon 90i microscope and the Neurolucida software (MicroBrightField). The tips of the cannulas were as the indicator of the injection sites on the section drawings and projected onto a representative section. The rat brain atlas of Paxinos and Watson (2007) was used to guide the delineation of ACC's borders.

## Results

### Histology

Two cannulas were implanted in ACC, one in each hemisphere, and their location was confirmed using Magnetic Resonance Imaging (MRI) scans and histological reconstructions. Figure 2 shows the placements of the cannulas on coronal sections, indicating that the injection sites were located in dorsal ACC, including Cg1, and Cg2 (Paxinos and Watson, 2007).

### Effects of D1 receptors blockade during the treatment phase

#### Qualitative observations

During the treatment phase, naïve rats received microinfusions of either SCH23390 or saline before daily testing sessions, in order to determine whether blockade of D1Rs in ACC impairs task acquisition. When placed in the testing apparatus after infusions, control rats typically alternate between lever contacts and visits to the food well, rest and self-grooming when behavior did not produce rewards in the early sessions. During observation, the observer typically alternates between exploration of the chamber, rest and interactions with the demonstrator through the wire mesh. Despite several training days without succeeding to earn rewards, control rats maintained interactions with the lever (see Figure S1).

Rats with SCH23390 microinfusions displayed a contrasting behavioral profile, with a poorer exploratory behavior in both TE-SCH and LeO-SCH compared to controls. When placed in the actor's compartment, they briefly explored the cage by sniffing the corners of the compartment (3–4 min), then took a rest position with active self-grooming for most of the remaining time. When first introduced in the testing compartment, SCH23390 infused rats approached the lever in the first sessions but were slower than controls (see Figure S1). With successive testing sessions, lever approach decreased drastically, with the 1st contact occurring with extremely high latencies. During the observation phase, LeO-SCH rats interacted less frequently with the actor, often turning their head away from it (see Figure S2).

During testing, free rewards were delivered occasionally in the food well. This served two purposes: one was to keep the animals motivated to explore and interact with the lever; the other was to assess their level of motivation for rewards, and their feeding behavior. Although, SCH23390 infused rats were generally slower to visit the food well than controls upon hearing the click of the food dispenser, there was no apparent lack of motivation for food nor any apparent feeding problems. When the animals were returned to their home cage, there were no apparent deficits in locomotor or feeding behavior. More importantly, control experiments on aversive conditioning revealed no food avoidance, whether in Saline or SCH23390 animals.

#### Quantitative results: general aspects

Multiple behavioral variables were scored (see Methods), and analyzed in order to assess learning rates and the effects of D1Rs blockade. The MANOVA revealed significant main effects of treatment [*F*_(6, 600)_ = 975.79, *P* < 0.001], learning type [*F*_(6, 600)_ = 107.66, *P* < 0.001], and session [*F*_(58, 600)_ = 16.29, *P* < 0.001]. There were also significant treatment × learning type [*F*_(6, 600)_ = 108.14, *P* < 0.001], treatment × session [*F*_(58, 600)_ = 16.54, *P* < 0.001], learning type × session [*F*_(58, 600)_ = 3.80, *P* < 0.001] and treatment × learning type × session [*F*_(58, 600)_ = 3.73, *P* < 0.001] interactions. The present analysis focuses on univariate ANOVAs of two key variables: the numbers of correct LPs and the latencies. For comparison of the numbers of sessions needed to reach criterion we used the non-parametric Mann-Whitney *U*-test.

#### Gain from observation in control groups

**Figure 3:**
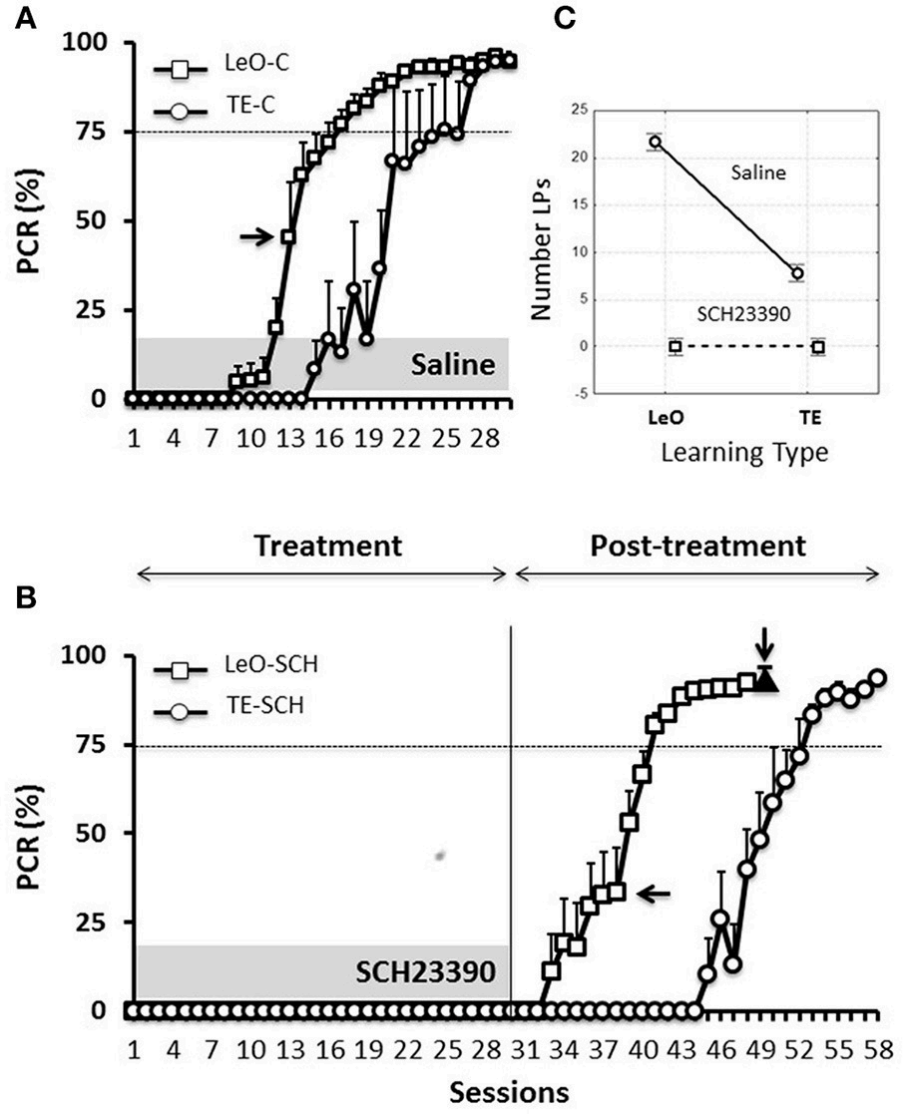
**Learning profiles in controls and experimental rats**. Learning is represented as the change in the percentage correct responses (PCR). **(A)** *Evolution of the PCR in control groups (saline injections)*. The symbols represent mean PCR for 6 rats, and the bars represent the standard error of the mean (sem). Triangles, trial-and-error (TE) group; Squares, observational learning (LeO) group. The dashed horizontal line represents the learning criterion (75%). Horizontal arrow points to the 1st session at which differences between groups start to be significant (Tukey HSD *post-hoc* test, *P* < 0.05). **(B)** *Evolution of the PCR in rats infused with SCH23390*. Same conventions as in **(A)**. The black triangle (indicated by the vertical arrow) represents the PCR for injections of SCH23390 at session 49, i.e., after learning. **(C)** *Treatment* × *learning type interaction, F*_(2, 599)_ = 108.14, *P* < 0001. The plot shows the mean number of lever pushes (LPs) plotted for each learning type (LeO vs. TE), as a function of treatment (Saline vs. SCH23390).

Figure 3A illustrates the results for the control animals, and it shows that learning occurred earlier following observation (LeO-C), compared to trial-and-error learning (TE-C). A univariate 3-way ANOVA (*treatment*: Saline vs. SCH23390; *learning type:* TE vs. LeO; *session:* 30) revealed a main effect of learning type on the number of rewarded LPs [*F*_(1, 600)_ = 215.68; *P* < 0.001; η^2^ = 0.05] and a significant treatment × learning type interaction (Figure 3C). *Post-hoc* analysis showed that observation elicited significantly more rewarded lever pushes (mean correct LPs over 30 sessions: LeO-C = 21.71 vs. TE-C = 7.78; *P* < 0.001). Importantly, observation accelerated learning as measured by the number of sessions needed to reach the learning criterion. Indeed, the mean number of sessions to criterion decreased significantly in the LeO-C group (mean number of sessions: LeO-C = 16.80 vs. TE-C = 22.00; *P* < 0.05, Mann-Whitney *U*-test). Thus, under the conditions of the present study, the observation of an expert conspecific performing the lever-push task to receive food reward led to faster learning (fewer sessions to criterion) and better performance (more LPs) than TE learning.

#### Effect of treatment: D1Rs blockade suppresses overt instrumental responding

Microinfusions of SCH23390 in ACC, unlike those of Saline, suppressed **responding** in both experimental groups (TE-SCH and LeO-SCH; Figures 3B,C). Analysis of the numbers of LPs showed a significant main effect of treatment [*F*_(1, 600)_ = 966.48; *P* < 0.001; η^2^ = 0.21] and significant interactions of treatment × session [*F*_(29, 600)_ = 38.74; *P* < 0.001; η^2^ = 0.24], treatment × learning type [*F*_(1, 600)_ = 215.68; *P* < 0.001; η^2^ = 0.05], and treatment × session × learning type [*F*_(29, 600)_ = 6.89; *P* < 0.001; η^2^ = 0.03]. *Post-hoc* analysis revealed a significant difference in mean LPs between experimental groups and their respective controls (TE-C = 7.78 vs. TE-SCH = 0.00, *P* < 0.001; LeO-C = 21.71 vs. LeO-SCH = 0.71; *P* < 0.001). In addition to the complete suppression of rewarded LPs, treatment affected all measured behavioral variables, including latencies (see below and Tables S1, S2).

Thus, at the end of the treatment phase, control rats had acquired the lever-push task, which they performed with more than 90% correct responding (Figure 3A), indicating that saline injections into ACC did not impair the rats' learning abilities. By contrast, rats infused with SC23390 expressed no **overt responding** throughout the 30 sessions of the treatment phase (Figure 3B).

### Post-treatment phase: covert learning spared by D1Rs blockade

At session 31, the treatment was ended, and the experimental rats were further tested during a post-treatment phase of 18 (LeO-SCH) or 28 sessions (TE-SCH). Figure 3B shows that the SCH23390 treated rats were able to learn during these post-treatment sessions, indicating that the repeated D1Rs blockade in ACC did not permanently impair the rats' ability to learn.

#### ACC dopamine D1 activity is not required for covert learning

The key question addressed here is whether SCH23390 treated rats covertly acquired the task through observation during the treatment phase, despite complete suppression of overt responding by the blockade of D1Rs. To examine this issue, we compared the performance across the groups during the first 18 sessions of the post-treatment phase (sessions 31–48) in LeO-SCH and TE-SCH groups and the first 18 sessions (sessions 1–18) of the control groups. Our specific hypothesis was that if SCH23390 treated rats (LeO-SCH) covertly learned through observation during the treatment phase, they would reach the criterion faster during the post-treatment phase compared to naïve rats injected with Saline (LeO-C).

**Figure 4:**
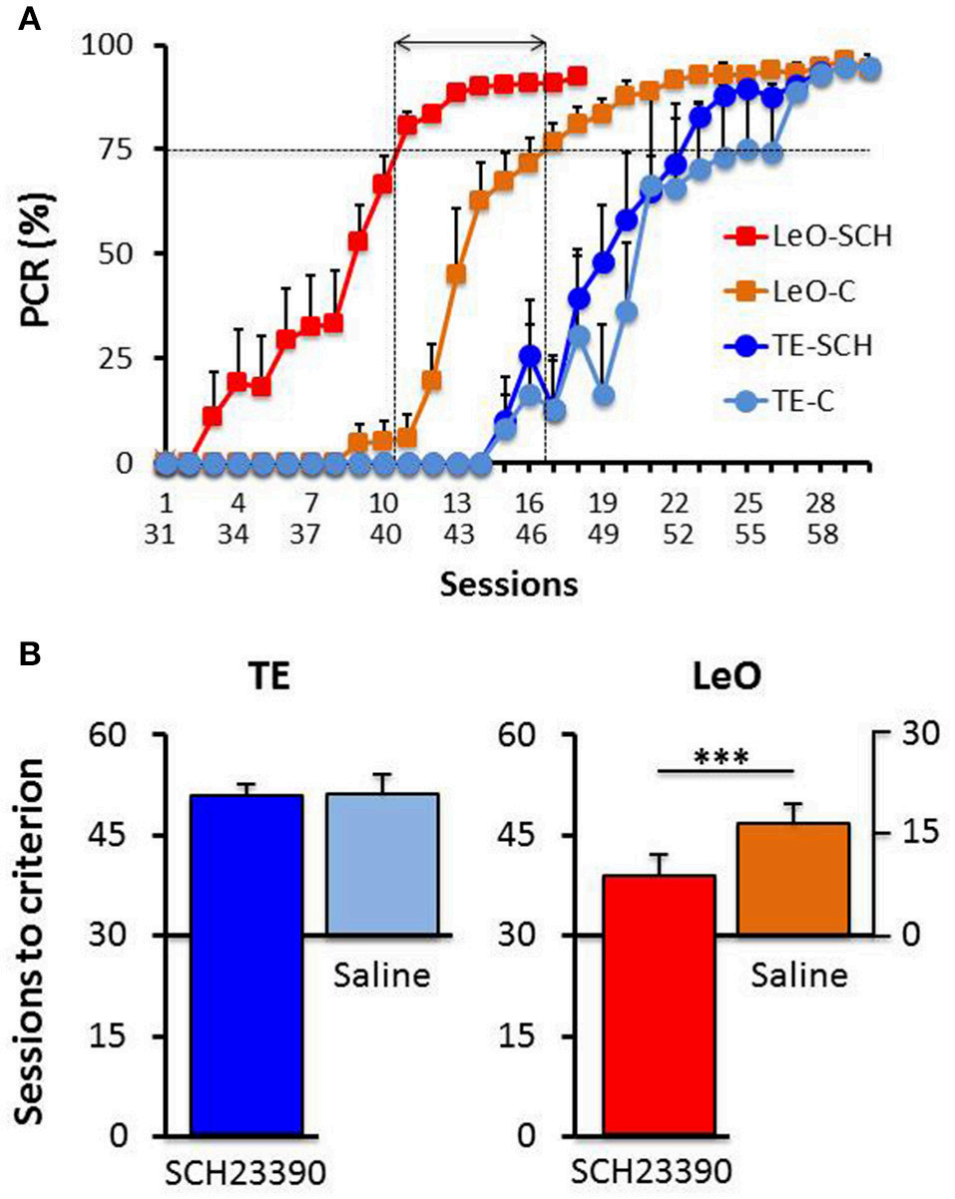
**Post-treatment learning: comparison with controls. (A)** *The PCR values are plotted as a function of sessions*, superimposed for the experimental groups TE-SCH (dark blue), LeO-SCH (red; sessions 31–58), and control groups TE-C (light blue) and LeO-C (orange; sessions 1–30). The vertical dotted lines depict the sessions at which learning criterion was reached by LeO-SCH (session 9) and LeO-C (session 16), and the horizontal arrow depicts the difference between the curves in terms of numbers of sessions (covert learning). **(B)** *Comparison of numbers of sessions to criterion*. Same color code as in **(A)** The mean numbers of sessions required to reach criterion (75% correct responses) are represented for controls (Saline; light blue and orange bars), and experimental groups (SCH23390; red and dark blue bars) for TE and LeO. Note that the y-axis for Saline groups is aligned with session 30 of the experimental groups. ^***^P < 0.001.

A univariate 3-way ANOVA (*treatment*: Saline, SCH23390; *learning type*: TE vs. LeO; *Session*: 18) of the number of rewarded LPs showed significant effects of treatment [*F*_(1, 360)_ = 68.01; *P* < 0.001; η^2^ = 0.03], session [*F*_(17, 360)_ = 45.81; *P* < 0.001; η^2^ = 0.34] and learning type [*F*_(1, 360)_ = 439.88; *P* < 0.001; η^2^ = 0.19] as well as treatment × session [*F*_(17, 360)_ = 2.68; *P* < 0.001; η^2^ = 0.02], treatment × learning type [*F*_(1, 360)_ = 52.31; *P* < 0.001; η^2^ = 0.02], session × learning type [*F*_(17, 360)_ = 29.87; *P* < 0.001; η^2^ = 0.22] and treatment × session × learning type [*F*_(17, 360)_ = 2.82; *P* < 0.001; η^2^ = 0.02] interactions. Importantly, *post-hoc* analysis showed that the number of correct LPs differed significantly across the observational groups (mean LPs: LeO-SCH = 11.56 vs. LeO-C = 5.63; *P* < 0.001), but not across the trial-and-error groups (mean LPs: TE-SCH = 0.75 vs. TE-C = 0.36; *P* = 0.89). Thus during the post-treatment phase (sessions 31–48), LeO-SCH animals, but not TE-SCH ones, performed better than naïve rats. In addition, as illustrated in Figure 4, LeO-SCH animals learned faster. Comparison of the numbers of sessions to criterion (Mann-Whitney *U*-test) revealed significant differences (mean number of sessions: LeO-SCH = 10.17 vs. LeO-C = 16.83; *P* = 0.0037), with a gain of nearly 7 sessions from observation during the treatment phase. By contrast, there was no significant difference between TE groups (mean number of sessions: TE-SCH = 20.67 vs. TE-C = 22.00; *P* = 0.124). These results indicate that LeO-SCH animals acquired information on the target instrumental behavior during the treatment phase, supporting the hypothesis that covert learning through observation was spared by blockade of D1Rs (Figures 3, 4).

#### Long-term effects of D1 receptor blockade

**Figure 5:**
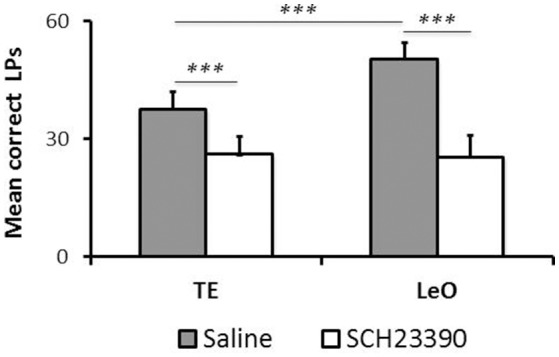
**Long-term effect of SCH23390**. The graph represents the mean numbers of correct lever presses (LPs) performed by each group after the learning criterion was reached (number of sessions = 6). ^***^ *P* < 0.001.

Although, SCH23390 treated animals mastered the instrumental task during the post-treatment testing, we noted that their performance remained low, relative to controls. To examine this aspect of their behavior, we compared the number of LPs across groups during the last sessions (*n* = 6), after all rats had reached the learning criterion. Figure 5 illustrates the results showing a strong and significant decrease in the rats' LP performance following SCH23390 treatment. A univariate repeated measures ANOVA revealed significant main effects of treatment [*F*_(1, 120)_ = 108.59; *P* < 0.001; η^2^ = 0.39], session [*F*_(5, 120)_ = 3.52; *P* < 0.01; η^2^ = 0.08], and learning type [*F*_(1, 120)_ = 11.70; *P* < 0.001; η^2^ = 0.04] as well as a treatment × learning type interaction [*F*_(1, 120)_ = 14.58; *P* < 0.001; η^2^ = 0.05]. *Post-hoc* analysis revealed significant differences in the numbers of LPs between experimental groups and controls (mean number of LPs: LeO-SCH = 25.53 vs. LeO-C = 50.39; *P* < 0.001; TE-SCH = 26.22 vs. TE-C = 37.75; *P* < 0.001). Interestingly, there was no significant difference between SCH23390 treated groups (*P* = 0.99), indicating that on a long term basis, the treatment had abolished the advantage of observational learning over trial-and-error learning alone.

#### Microinfusions after learning

In one group of rats (LeO-SCH), intra-ACC microinfusions of SCH23390 were made after they had mastered the task during the post-treatment phase, in order to evaluate the effects of D1Rs blockade on task execution after learning. The result of this experiment (Figure 3B, black triangle) shows that SCH23390 microinfusions had no effect, illustrating the selective effects of D1Rs blockade on task learning, rather than its execution once learned.

### Effect of D1Rs blockade on latencies of first lever contact

**Figure 6:**
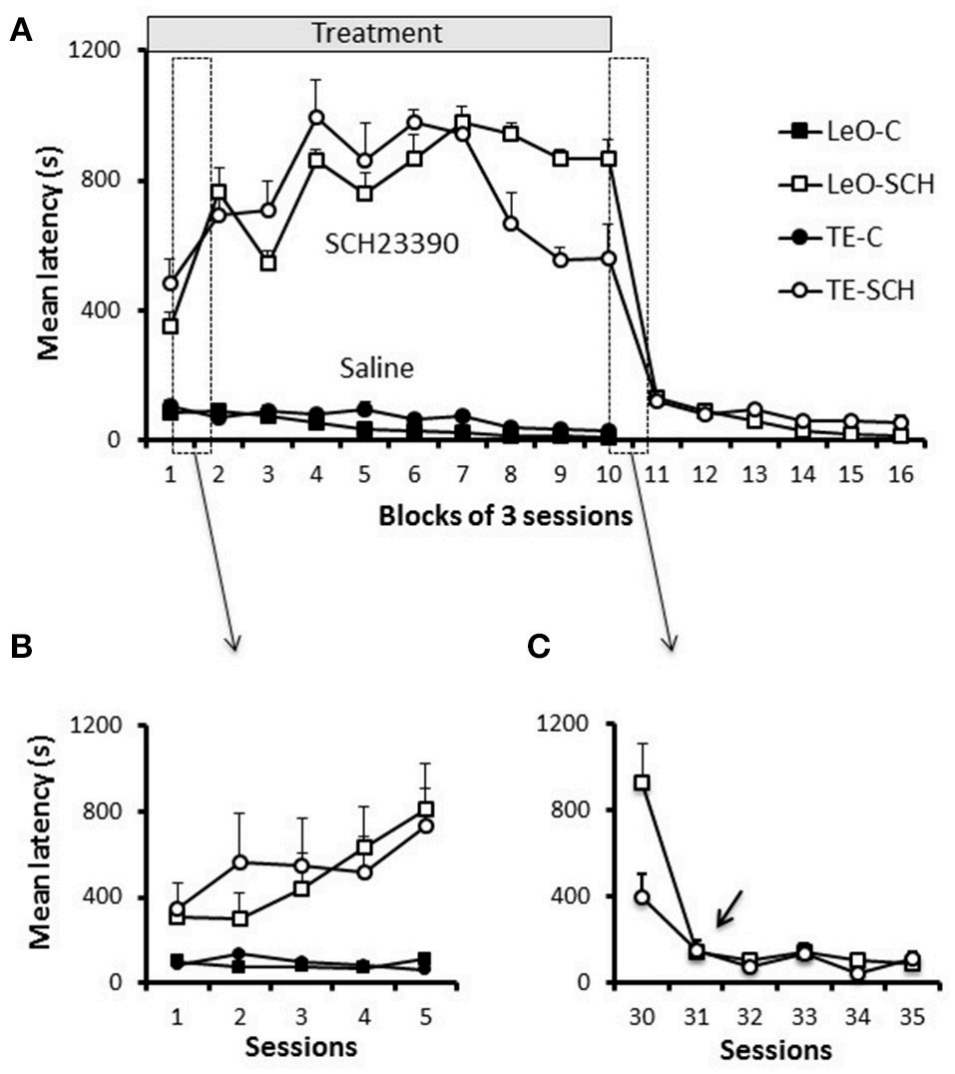
**Latencies. (A)** Latencies throughout training. Latencies of first lever contact across groups are plotted per blocks of 3 training sessions. Symbols represent average values across rats, and the bars show the standard error of the mean (sem). The first 5 sessions, which are included in the two boxes (dashed vertical rectangles), are shown in **(B,C)**. **(B)** Latencies during the first 5 sessions. **(C)** Latencies for 5 sessions of the transition period from treatment to post-treatment in the experimental groups (TE-SCH, LeO-SCH). The arrow shows that the decrease in latency depicted in **(A)**, between sessions 30 and 33, occurred at session 31.

One striking feature of SCH23390 treated rats was their reduced exploratory activity during testing, especially as assessed by lever pushing. We used the latency of the first lever contact in each session to investigate the vigor with which rats approached the lever when they were introduced in the testing apparatus. Figure 6A illustrates the general pattern of latencies and shows that experimental rats were strikingly slow to contact the lever, compared to controls. A univariate 3-way ANOVA revealed main effects of treatment [*F*_(1, 600)_ = 1006.45; *P* < 0.001; η^2^ = 0.55], session [*F*_(29, 600)_ = 2.35; *P* < 0.001; η^2^ = 0.04] and treatment × session interaction [*F*_(29, 600)_ = 2.76; *P* < 0.001; η^2^ = 0.04] but no significant effect of learning type (*P* = 0.94). A finer analysis of changes in latencies (Figure 6B) showed a progressive increase during the early sessions of training in SCH23390 treated rats; the animals were slow from the 1st session, but their state worsened during the next sessions and remained so throughout the treatment phase. Analysis of the total number of lever contacts (see Figure S1) confirmed the decline of the lever contacts within the early sessions in SCH23390 treated rats, which failed to maintain the lever-approach behavior required for learning. When microinjections of SCH23390 ended (Figure 6C), the latencies dropped sharply to the level of controls.

## Discussion

In this study, we have shown that blockade of D1Rs in ACC completely suppressed overt instrumental responding. Using observational learning, where the learner acquired knowledge pertaining to action-outcome associations without exerting any physical behavioral effort, we found that covert learning was left intact by D1Rs blockade. Because rats with blockade of D1Rs learned faster during the post-treatment testing than their respective controls, we conclude that dopamine D1 activity in ACC is not necessary for acquiring task-related knowledge. In addition, whereas rats recovered their full ability to learn during subsequent testing sessions (without D1R blockade), they displayed a long-lasting impairment in their willingness to lever-push for food. Blockade of D1Rs once the task was learned had no effect on performance. Overall, these findings suggest that ACC dopamine activity is not required for the formation of action-outcome associations. The results suggest that the suppression of responding reported here is a consequence of impaired regulation of effort tolerance. Rats with D1R blockade demonstrate a degree of effort intolerance that not only impairs their ability to learn associations through trial and error, but also to perform behaviors that depend on already learned associations.

### Specificity of the observed effects

A typical concern with pharmacological manipulations is the diffusion into neighboring areas. Although, the cannulas were centered in the ACC (Figure 2), the diffusion of the SCH23390 might have extended into adjacent areas, where the blockade of D1Rs might have contributed to the observed effects. There are two lines of evidence that militate against this interpretation of our results. First, the diffusion of the SCH23390 has been shown to remain within 1–2 mm around the injection site (Granon et al., 2000). Second, the functional specializations of adjacent areas that could have been affected by the SCH23390 would be unlikely to cause the observed effects. The ACC is bordered, ventrally and anteriorly by the prelimbic and infralimbic areas, and dorsolaterally by a premotor area sometimes called M2, but more properly considered as an agranular premotor area of unknown affinity to the premotor areas of primates. Prelimbic and infralimbic areas are major contributors of the frontal–basal ganglia limbic loop in control of behavior, but these areas seem to be more involved in regulating the balance between habits and goal-directed behavior than in response-reinforcer (action-outcome) learning *per se* (Ragozzino, 2000). There is no evidence that they contribute crucially to the effort-based decisions affected here. Furthermore, our injections were made at anteroposterior coordinates relative to the bregma (AP = 1.44 - AP = 1.80) that make it unlikely that the antagonist spread into either the prelimbic or infralimbic cortex. Spread of SCH23390 into the dorsally adjacent premotor area could explain the reduction in motor activity, but this is unlikely to be the main source of the observed effects given the implantation of the cannulas deep in the medial wall of the cortex. In addition, as stated in the results, the rats did not show motor deficits outside the testing apparatus.

### ACC dopamine activity and instrumental learning

As reviewed in the Introduction, several studies have investigated the role of ACC in learning using a variety of tasks (Bussey et al., 1997; Cardinal et al., 2002, 2003; Floresco and Magyar, 2006; McKee et al., 2010), and a widely accepted view is that ACC plays a role in action-outcome learning. However, the present findings and those of a previous study by Jonkman and Everitt (2009) suggest that impairments in instrumental learning following ACC lesions or intra-ACC pharmacological manipulation may not necessarily result from a specific deficit in the formation of action-outcome associations. Rather, learning impairments appear to be a consequence of alteration in other processes, such as aversive conditioning or effort-based functions. In their study, Jonkman and Everitt (2009) used postsession intra-ACC infusions of anisomycin, a protein-synthesis inhibitor assumed to affect local neuronal plasticity necessary for consolidation after a learning session. However, they demonstrated that infusions of anisomycin into ACC after consumption of sucrose pellets produced conditioned taste avoidance for sucrose pellets. When this effect on reward “liking” was dissociated from learning, Jonkman and Everitt (2009) found no selective effect on learning. They therefore concluded that ACC plasticity was not necessary for instrumental learning. In the present study, SCH23390 infusions did not affect reward “liking” and appetite, as assessed by free delivery of the same food reward during the treatment phase. Our finding that covert learning through observation did not depend on dopamine activity suggests that suppression of instrumental learning is not due to an impairment in learning *per se* (see below). The involved mechanisms may differ, but both studies (Jonkman and Everitt, 2009; present study) support the conclusion that ACC plasticity is not necessary for instrumental learning. However, another similar study by McKee et al. (2010), using a similar task with blockade of glutamatergic NMDA receptors in ACC found that learning impairment could not be explained by alteration in primary food motivation or appetite, motor or motivational factors. In line with the prevailing view, McKee et al. (2010) concluded that ACC is required for instrumental learning, but not for task performance after learning. One possible explanation for this apparent discrepancy is that different manipulations of intra-ACC activity might lead to different kinds of reorganization in neuronal activity, both locally within ACC and in its interactions with the basal ganglia and other cortical areas (Zahm, 2000; Haber and Knutson, 2010).

### ACC dopamine, behavioral activation, and effort costs

The debate on whether motivation, motor functions, reward and learning are processed separately by specific subnetworks of the frontostriatal system has inspired a large number of empirical studies seeking to dissociate these processes one from another (see Salamone et al., 2007; Salamone, 2009; Kurniawan et al., 2011 for reviews;). Because dopamine in the limbic circuit has functions at the intersection of emotional, motivational, cognitive and motor functions, dissociating these processes is challenging. Recent reviews have offered integrative hypotheses of the multiple functions of mesocortical and mesolimbic dopamine (e.g., Salamone, 2009; Kurniawan et al., 2011; Berridge and O'Doherty, 2013; Salamone et al., 2016). They emphasized the role of dopamine in effortful behavior, behavioral activation, vigor and pathologies characterized by the lack of vigor, such as apathy (Kurniawan et al., 2011), anergia, abulia and depression, among others (Salamone et al., 2016). For example, Salamone (2009) suggested behavioral activation as a key role of mesolimbic dopamine, to refer to “the vigor, persistence and effort seen in the pursuit of motivational stimuli, and the heightened activity induced by conditioned stimuli that predict reinforcers.” Niv et al. (2007) have proposed a model of vigor of action, as the inverse of latency, where different dopamine functions are taken into account, namely learning (Schultz et al., 1997; Schultz and Dickinson, 2000), incentive salience (Berridge and Robinson, 1998) and behavioral activation (Salamone et al., 2007; Salamone, 2009). Finally, Kurniawan et al. (2011) summarized evidence from a wide range of studies to suggest that the pathology of effortful actions in humans is apathy, the most severe form of which is auto-activation deficit (van Reekum et al., 2005) characterized by an inability to internally generate goal oriented actions associated with lesions of basal ganglia and prefrontal cortex, especially ACC (van Reekum et al., 2005; Levy and Dubois, 2006; Passingham et al., 2010).

Multiple indicators in the present study suggest that a lack of vigor, more formally known as effort tolerance, and a deficit in behavioral activation may account for the complete suppression of instrumental responding reported here. First, an alteration in basic motor functions does not explain the results. On the one hand, SCH23390 treated rats displayed normal locomotion and feeding behavior in their home cage and consumed regular food pellets. However, they displayed no effort to gain access to tasty food in the testing apparatus in that they did not perform a single rewarded lever push, although they interacted with the lever in the initial phase of training (see Figure S1). On the other hand, SCH23390 microinfusions after learning had no effect on performance, suggesting that dopamine D1Rs blockade did not alter the rats' motor ability to push the lever, at least independently of learning. Second, suppression of overt learning is unlikely to have resulted from alteration in primary motivation for food reward or in the animals' appetite or from altered feeding behavior, as assessed by free-reward testing. SCH23390 infused rats were able to consume free rewards, and a control experiment (see Supplementary Results) confirmed that dopamine blockade in ACC did not reduce the incentive value of food rewards. Finally, recovery after the treatment was partial: rats recovered full learning abilities, but their willingness to work for food remained low after the task was mastered.

Taken together, the most parsimonious explanation of the suppression of responding by the blockade of dopamine D1 activity in ACC in the present study is an impairment in regulating effort tolerance and what might be called “energy economics.” As addressed in the results section (Figure 6), a key feature in SCH23390 infused rats seemed to lack the expenditure of energy necessary for instrumental responding oriented to food reward. This conclusion agrees with previous reports showing that lesions, inactivation or dopamine D1Rs blockade in ACC reduce effort tolerance in order to obtain higher reward (Walton et al., 2003; Schweimer and Hauber, 2006; Hosking et al., 2014). The present study further suggests that repeated D1Rs blockade might cause long lasting impairment of effort-based behavior, perhaps including both physical and cognitive effort (see below).

### Dopamine circuits of behavioral activation, effort, and costs

Pioneering studies in the 1980s have shown that DA antagonists or nucleus accumbens dopamine depletions suppress appetitive behavioral activities (reviewed in Salamone et al., 2007). Dopamine has been thought, since these early rodent studies, to regulate the willingness to produce effortful behavior for reward, and a number of studies have been dedicated to the role of nucleus accumbens (NAc) dopamine as a central node within a circuit that regulates behavioral activation (e.g., Salamone and Correa, 2012; Salamone et al., 2012, 2016). ACC is part of this corticostriatal limbic circuit that also includes the basolateral nucleus of the amygdala (BLA; Floresco and Ghods-Sharifi, 2007) and the dorsomedial striatum (McKee et al., 2010). Other studies have pointed to interactions among the components of this circuit. For example, it has been shown that effort-based decisions are mediated by a serial transfer of information between the BLA and ACC (Floresco and Ghods-Sharifi, 2007) and between NAc and ACC (Hauber and Sommer, 2009). Thus, blockade of D1Rs in ACC in our experiment likely altered effort-related functions via these interactions among ACC, BLA and NAc (see for reviews Kurniawan et al., 2011; Floresco, 2015), leading to a level of effort intolerance that blocked instrumental learning.

### Learning, cognitive, and physical effort

Other studies that have manipulated intra-ACC activity before or during acquisition of operant tasks (Jonkman and Everitt, 2009; McKee et al., 2010) have also reported a severe impairment of instrumental responding. By contrast, when the animals were required to choose between previously learned options that differ in terms of effort costs (Walton et al., 2003; Schweimer et al., 2005; Schweimer and Hauber, 2006; Hosking et al., 2014), blockade of D1Rs resulted in only a relative impairment in performance.

There has been controversy about whether effort intolerance reflects physical effort, cognitive effort or both (Walton et al., 2003; Schweimer et al., 2005; Floresco et al., 2006; Salamone et al., 2007, 2016; Braver et al., 2014; Holec et al., 2014; Hosking et al., 2015; Westbrook and Braver, 2015, 2016). If we limit the discussion to the role of intra-ACC dopamine to effort processes, the present results seem to favor a specific role in physical effort, as physical responding was impaired by blockade of D1Rs, but not acquisition of task-relevant information through observation (cognitive effort). However, cognitive effort might not be limited to information processing *per se*, but would include the willingness to engage in effortful behavior. ACC dopamine activity is clearly necessary for engaging in effortful behavior, a hypothesis which is supported by a recent simulation study (Holroyd and McClure, 2015) where ACC occupies a hierarchical position allowing it to control choices between behavioral options. If options are taken as “to learn” or “to quit,” our rats with blockade of D1Rs seem to have selected the latter. In line with this result, the simulation study of Holroyd and McClure (2015) predicts that disruption of dopamine processing in ACC results in a withdrawal of control over effortful behavior.

### Long-term plasticity in the effort-based functions

Although, rats with SCH23390 microinfusions in ACC recovered instrumental learning abilities during the post-treatment phase, they did not fully recover a normal effort tolerance. Indeed, despite perfect mastery of the task (>90% correct LPs), SCH23390 infused rats performed significantly fewer trials than controls. This long lasting reduction in the animals' willingness to perform the instrumental task for food reward, after learning, is likely to have resulted from some form of long-term plasticity within the limbic dopamine circuits. Repeated blockade of D1Rs in ACC appears to have permanently modified these circuits, possibly through a reduction in the sensitivity and/or density of D1Rs in ACC. In particular, the observed long-lasting reduction in task performance in the current study may reflect long-term changes in the ACC glutamatergic projection to NAc, leading to a reduction in activating motivational processes and effort-based functions (e.g., Kurniawan et al., 2011; Floresco, 2015). Future work might address the molecular mechanisms of this phenomenon.

### ACC dopamine, social behavior, and social learning

Although the neuronal mechanisms of social learning, including observational learning, are only beginning to be investigated by neuroscientists, available evidence suggests that this type of learning may rely on the same brain mechanisms as associative learning. In particular, neuroimaging studies in humans suggest that observational learning activates the brain networks involved in reinforcement-based learning (Behrens et al., 2008; Monfardini et al., 2008, 2013; Bellebaum et al., 2010, 2012; Burke et al., 2010; Kobza and Bellebaum, 2015), in addition to the network involved in action observation (Monfardini et al., 2013). Electrophysiological studies in humans have also shown that the feedback-related negativity (FRN), a presumably dopamine-related wave recorded in humans at the level of ACC in synchrony with errors, is modulated during observational learning (see Walsh and Anderson, 2012 for review). This is in line with human and non-human primate reports showing that damage of the prefrontal cortex, and more specifically of the ACC (Hadland et al., 2003; Rudebeck et al., 2006) disrupts social behavior. Furthermore, neuronal recordings in monkeys have shown that neurons in the prefrontal cortex and basal ganglia code the social value of reward (Chang et al., 2013), and monitor others' actions and their outcomes (Yoshida et al., 2011, 2012; Azzi et al., 2012; Hosokawa and Watanabe, 2012, 2015). Importantly, ACC appears to represent a node for the processing of both self and other's reward during decision-making tasks (Chang et al., 2013). These studies provide indirect evidence suggesting that dopamine activity in ACC is involved in social behavior, including social learning. Although, the present study did not address specifically social behavior, there were clear changes at the gross level following microinfusions of SCH23390 in ACC. In particular, treated rats systematically avoided social contact and interactions with the demonstrator during testing. However, if ACC plays a role in social behavior, intra-ACC dopamine D1 activity does not appear to be required for the acquisition of action-outcome association through observational learning. Perhaps ACC dopamine is involved in general purpose social mechanisms that also require effort-based functions, in line with the widely held view that ACC plays a role in the processing of social and emotional information. A coherent interpretation is that ACC dopamine activity may not be required for the acquisition of action-outcome associations, let it be through individual experience or through others' experience, but it plays an indirect role in both types of learning by regulating the invigorating function that translates anticipation of rewarding/pleasant outcomes into effortful instrumental actions, or social interactions with conspecifics.

### Limitations

There are at least three limitations that require some attention. One relates to the testing protocol. As TE and LeO animals were tested on overt learning with different time delays after microinfusions (TE rats were tested directly after microinfusions, whereas LeO rats were first tested on covert learning, then on overt learning) it might be argued that this aspect might explain the observed differences between TE-SCH and LeO-SCH groups. For example, what if SCH23390 were more (or less) effective in blocking D1Rs during the first 20 min than during the later period. This is unlikely for at least two reasons. On one hand, previous studies have shown that, using the same doses and concentrations as we have used, the effects of SCH23390 last for 40–60 min in the cerebral cortex (Hietala et al., 1992). On the other hand, both TE-SCH and LeO-SCH rats were severely impaired during the overt testing phase. Interestingly, our claim that covert learning is not impaired is strengthened by the fact that observation occurred within the first 20 min following SCH23390 microinfusions, i.e., the same phase where overt learning was severely impaired in TE-SCH rats.

Another limitation relates to the comparison of post-treatment learning in LeO-SCH group with learning in LeO-C group under saline microinfusions. It could be argued that the advantage of LeO-SCH animals over LeO-C animals, which we have interpreted as the spared covert learning (through observation), might be explained by the fact that LeO-SCH rats were not injected during the post-treatment phase. Handling during the injections and the induced stress might indeed explain partly the slower learning in LeO-C compared to LeO-SCH animals. While this possibility cannot be ruled out, the stress level is unlikely to explain the observed differences because the animals have been habituated for several daily sessions. One way to eliminate this limitation would have been to use a new control group, without handling or infusions, to compare post-treatment learning in LeO-SCH rats with naïve rats. However, this would be problematic for another reason: LeO-SCH animals received microinfusions over 30 sessions, with the resulting brain damage, whereas the control rats would be brain-damage free. Taken together, and given the limited scientific added value of a new control group, there is an ethical advantage of re-using LeO-C as a control group.

Finally, comparison between TE and LeO groups of rats requires explanation. If our goal were to strictly compare TE and LeO learning, we would have used additional controls to eliminate alternative interpretations of some of the observed differences. In particular, one could argue that the mere experience of being in the observational chamber might be the source of the observed differences between LeO and TE groups. This possibility can only be ruled out by placing a group of TE-SCH rats in the observation chamber, expose them to an identical number of correctly performed trials but without an expert demonstrator (e.g., computer controlled lever pushes). However, as our primary aim was to investigate how blockade of D1Rs affects two learning modalities, one with and one without prior exposition to a demonstration, the experimental design emphasized intra-learning type comparison (SCH vs. Saline).

## Author contributions

MA, DB, and PC developed the study concept and design. MA, DB, MM, and SS performed surgical implantations. Animal testing was performed by MA, MH, and PC. Data analyses were performed by MA, PC, ME, and DB; MA and SS performed the histology work. Finally, DB, PC, MM, ME, and MA wrote the paper and all authors provided critical revisions. All authors approved the final version of the paper for submission.

### Conflict of interest statement

The authors declare that the research was conducted in the absence of any commercial or financial relationships that could be construed as a potential conflict of interest. The reviewer SP and handling Editor declared their shared affiliation, and the handling Editor states that the process nevertheless met the standards of a fair and objective review.

## References

[B1] Amiez C. Joseph J. P. Procyk E. (2006). Reward encoding in the monkey anterior cingulate cortex. Cereb. Cortex 16, 1040–1055. 10.1093/cercor/bhj046 16207931 PMC1913662

[B2] Azzi J. C. B. Sirigu A. Duhamel J. R. (2012). Modulation of value representation by social context in the primate orbitofrontal cortex. Proc. Natl. Acad. Sci. U.S.A. 109, 2126–2131. 10.1073/pnas.1111715109 22308343 PMC3277550

[B3] Behrens T. E. J. Hunt L. T. Woolrich M. W. Rushworth M. F. S. (2008). Associative learning of social value. Nature 456, 24524–24529. 10.1038/nature07538 19005555 PMC2605577

[B4] Bellebaum C. Jokisch D. Gizewski E. R. Forsting M. Daum I. (2012). The neural coding of expected and unexpected monetary performance outcomes: dissociations between active and observational learning. Behav. Brain Res. 227, 241–251. 10.1016/j.bbr.2011.10.042 22074898

[B5] Bellebaum C. Kobza S. Thiele S. Daum I. (2010). It was not my fault: Event-related brain potentials in active and observational learning from feedback. Cereb. Cortex 20, 2874–2883. 10.1093/cercor/bhq038 20308202

[B6] Berridge K. C. O'Doherty J. P. (2013). From Experienced Utility to Decision Utility. London: Elsevier Inc.

[B7] Berridge K. C. Robinson T. E. (1998). What is the role of dopamine in reward: Hedonic impact, reward learning, or incentive salience? Brain Res. Rev. 28, 309–369. 10.1016/S0165-0173(98)00019-8 9858756

[B8] Bissonette G. B. Roesch M. R. (2015). Neural correlates of rules and conflict in medial prefrontal cortex during decision and feedback epochs. Front. Behav. Neurosci. 9:266. 10.3389/fnbeh.2015.00266 26500516 PMC4594023

[B9] Braver T. S. Krug M. K. Chiew K. S. Kool W. Westbrook J. A. Clement N. J.. (2014). Mechanisms of motivation-cognition interaction: challenges and opportunities. Cogn. Affect. Behav. Neurosci. 14, 443–72. 10.3758/s13415-014-0300-0 24920442 PMC4986920

[B10] Brosnan S. F. de Waal F. B. M. (2004). Socially learned preferences for differentially rewarded tokens in the brown capuchin monkey (*Cebus apella*). J. Comp. Psychol. 118, 133–139. 10.1037/0735-7036.118.2.133 15250800

[B11] Burke C. J. Tobler P. N. Baddeley M. Schultz W. (2010). Neural mechanisms of observational learning. Proc. Natl. Acad. Sci. U.S.A. 107, 14431–14436. 10.1073/pnas.1003111107 20660717 PMC2922583

[B12] Bussey T. J. Everitt B. J. Robbins T. W. (1997). Dissociable effects of cingulate and medial frontal cortex lesions on stimulus-reward learning using a novel Pavlovian autoshaping procedure for the rat: Implications for the neurobiology of emotion. Behav. Neurosci. 111, 908–919. 10.1037/0735-7044.111.5.908 9383513

[B13] Bussey T. J. Muir J. L. Everitt B. J. Robbins T. W. (1996). Dissociable effects of anterior and posterior cingulate cortex lesions on the acquisition of a conditional visual discrimination: facilitation of early learning vs. impairment of late learning. Behav. Brain Res. 82, 45–56. 10.1016/S0166-4328(97)81107-2 9021069

[B14] Cardinal R. N. Parkinson J. A. Marbini H. D. Toner A. J. Bussey T. J. Robbins T. W.. (2003). Role of the anterior cingulate cortex in the control over behavior by Pavlovian conditioned stimuli in rats. Behav. Neurosci. 117, 566–587. 10.1037/0735-7044.117.3.566 12802885

[B15] Cardinal R. N. Parkinson J. A. Lachenal G. Halkerston K. M. Rudarakanchana N. Hall J.. (2002). Effects of selective excitotoxic lesions of the nucleus accumbens core, anterior cingulate cortex, and central nucleus of the amygdala on autoshaping performance in rats. Behav. Neurosci. 116, 553–567. 10.1037/0735-7044.116.4.553 12148923

[B16] Chang S. W. C. Gariépy J.-F. Platt M. L. (2013). Neuronal reference frames for social decisions in primate frontal cortex. Nat. Neurosci. 16, 243–250. 10.1038/nn.3287 23263442 PMC3557617

[B17] Coles M. G. H. Scheffers M. K. Holroyd C. (1998). Berger's dream? The error-related negativity and modern cognitive psychophysiology, in Quantitative and Topological EEG and EMG Analysis, eds, Witte H. Zwiener U. Schack B. Döring A. (Jena-Erlangen: Druckhaus Mayer Verlag), 96–102.

[B18] Cousins M. S. Salamone J. D. (1994). Nucleus accumbens dopamine depletions in rats affect relative response allocation in a novel cost/benefit procedure. Pharmacol. Biochem. Behav. 49, 85–91. 10.1016/0091-3057(94)90460-X 7816895

[B19] Floresco S. B. (2015). The nucleus accumbens: an interface between cognition, emotion, and action. Annu. Rev. Psychol. 66, 25–52. 10.1146/annurev-psych-010213-115159 25251489

[B20] Floresco S. B. Block A. E. Tse M. T. L. (2008). Inactivation of the medial prefrontal cortex of the rat impairs strategy set-shifting, but not reversal learning, using a novel, automated procedure. Behav. Brain Res. 190, 85–96. 10.1016/j.bbr.2008.02.008 18359099

[B21] Floresco S. B. Ghods-Sharifi S. (2007). Amygdala-prefrontal cortical circuitry regulates effort-based decision making. Cereb. Cortex 17, 251–260. 10.1093/cercor/bhj143 16495432

[B22] Floresco S. B. Magyar O. (2006). Mesocortical dopamine modulation of executive functions: beyond working memory. Psychopharmacology (Berl). 188, 567–585. 10.1007/s00213-006-0404-5 16670842

[B23] Floresco S. B. Magyar O. Ghods-Sharifi S. Vexelman C. Tse M. T. L. (2006). Multiple dopamine receptor subtypes in the medial prefrontal cortex of the rat regulate set-shifting. Neuropsychopharmacology 31, 297–309. 10.1038/sj.npp.1300825 16012531

[B24] Gabriel M. Kubota Y. Sparenborg S. Straube K. Vogt B. A. (1991). Effects of cingulate cortical lesions on avoidance learning and training induced unit activity in rabbits. Exp. Brain Res. 86, 585–600. 10.1007/bf00230532 1761092

[B25] Gehring W. J. Goss B. Coles M. G. H. Meyer D. E. Donchin E. (1993). A neural system for error detection and compensation. Psychol. Sci. 4, 385–390. 10.1111/j.1467-9280.1993.tb00586.x

[B26] Granon S. Passetti F. Thomas K. L. Dalley J. W. Everitt B. J. Robbins T. W. (2000). Enhanced and impaired attentional performance after infusion of D1 dopaminergic receptor agents into rat prefrontal cortex. J. Neurosci. 20, 1208–1215. 10648725 10.1523/JNEUROSCI.20-03-01208.2000 PMC6774157

[B27] Haber S. N. Knutson B. (2010). The reward circuit: linking primate anatomy and human imaging. Neuropsychopharmacology 35, 4–26. 10.1038/npp.2009.129 19812543 PMC3055449

[B28] Hadland K. A. Rushworth M. F. S. Gaffan D. Passingham R. E. (2003). The effect of cingulate lesions on social behaviour and emotion. Neuropsychologia 41, 919–931. 10.1016/S0028-3932(02)00325-1 12667528

[B29] Hauber W. Sommer S. (2009). Prefrontostriatal circuitry regulates effort-related decision making. Cereb. Cortex 19, 2240–2247. 10.1093/cercor/bhn241 19131436

[B30] Hayden B. Y. Heilbronner S. R. Pearson J. M. Platt M. L. (2011). Surprise signals in anterior cingulate cortex: neuronal encoding of unsigned reward prediction errors driving adjustment in behavior. J. Neurosci. 31, 4178–4187. 10.1523/JNEUROSCI.4652-10.2011 21411658 PMC3070460

[B31] Heyes C. M. Dawson G. R. (1990). A demonstration of observational learning in rats using a bidirectional control. Q. J. Exp. Psychol. B. 42, 59–71. 2326494

[B32] Hietala J. Sepp T. Lappalainen J. Syv E. (1992). Quantification of SCH 39166, a novel selective D1 dopamine receptor antagonist, in rat brain and *Blood* 23390, 455–458. 10.1007/BF02244814 1349751

[B33] Holec V. Pirot H. L. Euston D. R. (2014). Not all effort is equal: the role of the anterior cingulate cortex in different forms of effort-reward decisions. Front. Behav. Neurosci. 8:12. 10.3389/fnbeh.2014.00012 24478659 PMC3904092

[B34] Holroyd C. B. Coles M. G. H. (2002). The neural basis of human error processing: reinforcement learning, dopamine, and the error-related negativity. Psychol. Rev. 109, 679–709. 10.1037/0033-295X.109.4.679 12374324

[B35] Holroyd C. B. McClure S. M. (2015). Hierarchical control over effortful behavior by rodent medial frontal cortex: a computational model. Psychol. Rev. 122, 54–83. 10.1037/a0038339 25437491

[B36] Holroyd C. B. Nieuwenhuis S. Yeung N. Nystrom L. Mars R. B. Coles M. G. H.. (2004). Dorsal anterior cingulate cortex shows fMRI response to internal and external error signals. Nat. Neurosci. 7, 497–498. 10.1038/nn1238 15097995

[B37] Holroyd C. Yeung N. (2011). An integrative theory of anterior cingulate cortex function: option selection in hierarchical reinforcement learning. Neural Basis Motiv. 16, 333–349. 10.7551/mitpress/9780262016438.003.0018

[B38] Hosking J. G. Cocker P. J. Winstanley C. A. (2014). Dissociable contributions of anterior cingulate cortex and basolateral amygdala on a rodent cost/benefit decision-making task of cognitive effort. Neuropsychopharmacology 39, 1558–1567. 10.1038/npp.2014.27 24496320 PMC4023153

[B39] Hosking J. G. Floresco S. B. Winstanley C. A. (2015). Dopamine antagonism decreases willingness to expend physical, but not cognitive, effort: a comparison of two rodent cost/benefit decision-making tasks. Neuropsychopharmacology 40, 1005–1015. 10.1038/npp.2014.285 25328051 PMC4330516

[B40] Hosokawa T. Watanabe M. (2012). Prefrontal neurons represent winning and losing during competitive video shooting games between monkeys. J. Neurosci. 32, 7662–7671. 10.1523/JNEUROSCI.6479-11.2012 22649245 PMC6703568

[B41] Hosokawa T. Watanabe M. (2015). Egalitarian reward contingency in competitive games and primate prefrontal neuronal activity. Front. Neurosci. 9:165. 10.3389/fnins.2015.00165 26029039 PMC4432669

[B42] Isbaine F. Demolliens M. Belmalih A. Brovelli A. Boussaoud D. (2015). Learning by observation in the macaque monkey under high experimental constraints. Behav. Brain Res. 289, 141–148. 10.1016/j.bbr.2015.04.029 25934491

[B43] Jonkman S. Everitt B. J. (2009). Post-learning infusion of anisomycin into the anterior cingulate cortex impairs instrumental acquisition through an effect on reinforcer valuation. Learn. Mem. 16, 706–713. 10.1101/lm.1497709 19864297 PMC2775517

[B44] Kawai T. Yamada H. Sato N. Takada M. Matsumoto M. (2015). Roles of the lateral habenula and anterior cingulate cortex in negative outcome monitoring and behavioral adjustment in nonhuman primates. Neuron 88, 792–804. 10.1016/j.neuron.2015.09.030 26481035

[B45] Kennerley S. W. Walton M. E. Behrens T. E. J. Buckley M. J. Rushworth M. F. S. (2006). Optimal decision making and the anterior cingulate cortex. Nat. Neurosci. 9, 940–947. 10.1038/nn1724 16783368

[B46] Kobza S. Bellebaum C. (2015). Processing of action- but not stimulus-related prediction errors differs between active and observational feedback learning. Neuropsychologia 66, 75–87. 10.1016/j.neuropsychologia.2014.10.036 25446969

[B47] Kurniawan I. T. Guitart-Masip M. Dolan R. J. (2011). Dopamine and effort-based decision making. Front Neurosci. 5:81. 10.3389/fnins.2011.00081 21734862 PMC3122071

[B48] Levy R. Dubois B. (2006). Apathy and the functional anatomy of the prefrontal cortex-basal ganglia circuits. Cereb. Cortex 16, 916–928. 10.1093/cercor/bhj043 16207933

[B49] McKee B. L. Kelley A. E. Moser H. R. Andrzejewski M. E. (2010). Operant learning requires NMDA-receptor activation in the anterior cingulate cortex and dorsomedial striatum, but not in the orbitofrontal cortex. Behav. Neurosci. 124, 500–509. 10.1037/a0020270 20695649

[B50] Meunier M. Monfardini E. Boussaoud D. (2007). Learning by observation in rhesus monkeys. Neurobiol. Learn. Mem. 88, 243–248. 10.1016/j.nlm.2007.04.015 17572114

[B51] Monfardini E. Brovelli A. Boussaoud D. Takerkart S. Wicker B. (2008). I learned from what you did: retrieving visuomotor associations learned by observation. Neuroimage 42, 1207–1213. 10.1016/j.neuroimage.2008.05.043 18588987

[B52] Monfardini E. Gaveau V. Boussaoud D. Hadj-Bouziane F. Meunier M. (2012). Social learning as a way to overcome choice-induced preferences? Insights from humans and rhesus macaques. Front. Neurosci. 6:127. 10.3389/fnins.2012.00127 22969703 PMC3432509

[B53] Monfardini E. Gazzola V. Boussaoud D. Brovelli A. Keysers C. Wicker B. (2013). Vicarious neural processing of outcomes during observational learning. PLoS ONE 8:e73879. 10.1371/journal.pone.0073879 24040104 PMC3764021

[B54] Nieuwenhuis S. Holroyd C. B. Mol N. Coles M. G. H. (2004). Reinforcement-related brain potentials from medial frontal cortex: Origins and functional significance. Neurosci. Biobehav. Rev. 28, 441–448. 10.1016/j.neubiorev.2004.05.003 15289008

[B55] Niv Y. Daw N. D. Joel D. Dayan P. (2007). Tonic dopamine: Opportunity costs and the control of response vigor. Psychopharmacology (Berl). 191, 507–520. 10.1007/s00213-006-0502-4 17031711

[B56] Noudoost B. Moore T. (2011). Control of visual cortical signals by prefrontal dopamine. Nature 474, 372–375. 10.1038/nature09995 21572439 PMC3117113

[B57] Parkinson J. Willoughby P. Robbins T. Everitt B. (2000). Disconnection of the anterior cingulate cortex and nucleus accumbens core impairs Pavlovian approach behavior: further evidence for limbic cortical-ventral striatopallidal systems. Behav. Neurosci. 114, 42–63. 10.1037/0735-7044.114.1.42 10718261

[B58] Passingham R. E. Bengtsson S. L. Lau H. C. (2010). Medial frontal cortex: from self-generated action to reflection on one's own performance. Trends Cogn. Sci. 14, 16–21. 10.1016/j.tics.2009.11.001 19969501 PMC2806969

[B59] Paxinos G. Watson C. (2007). The Rat Brain in Stereotaxic Coordinates. San Diego, CA: Academic Press.

[B60] Puig M. V. Miller E. K. (2012). The role of prefrontal dopamine D1 receptors in the neural mechanisms of associative learning. Neuron 74, 874–886. 10.1016/j.neuron.2012.04.018 22681691 PMC3819197

[B61] Puig M. V. Miller E. K. (2014). Neural substrates of dopamine D2 receptor modulated executive functions in the monkey prefrontal cortex. Cereb. Cortex 25, 2980–2987. 10.1093/cercor/bhu096 24814093 PMC4537442

[B62] Quilodran R. Rothé M. Procyk E. (2008). Behavioral shifts and action valuation in the anterior cingulate cortex. Neuron 57, 314–325. 10.1016/j.neuron.2007.11.031 18215627

[B63] Ragozzino M. E. (2000). The contribution of cholinergic and dopaminergic afferents in the rat prefrontal cortex to learning, memory, and attention. Psychobiology 28, 238–247. 10.3758/BF03331982

[B64] Ragozzino M. E. Rozman S. (2007). The effect of rat anterior cingulate inactivation on cognitive flexibility. Behav. Neurosci. 121, 698–706. 10.1037/0735-7044.121.4.698 17663595

[B65] Rudebeck P. H. Buckley M. J. Walton M. E. Rushworth M. F. S. (2006). A role for the macaque anterior cingulate gyrus in social valuation. Science 313, 1310–1312. 10.1126/science.1128197 16946075

[B66] Rushworth M. F. S. Behrens T. E. J. Rudebeck P. H. Walton M. E. (2007). Contrasting roles for cingulate and orbitofrontal cortex in decisions and social behaviour. Trends Cogn. Sci. 11, 168–176. 10.1016/j.tics.2007.01.004 17337237

[B67] Rushworth M. F. S. Hadland K. Gaffan D. Passingham R. E. (2003). The effect of cingulate cortex lesions on task switching and working memory. J. Cogn. Neurosci. 15, 338–353. 10.1162/089892903321593072 12729487

[B68] Salamone J. D. (2009). Dopamine, behavioral economics, and effort. Front. Behav. Neurosci. 3:13. 10.3389/neuro.08.013.2009 19826615 PMC2759361

[B69] Salamone J. D. Correa M. (2012). The mysterious motivational functions of mesolimbic dopamine. Neuron 76, 470–485. 10.1016/j.neuron.2012.10.021 23141060 PMC4450094

[B70] Salamone J. D. Correa M. Farrar A. Mingote S. M. (2007). Effort-related functions of nucleus accumbens dopamine and associated forebrain circuits. Psychopharmacology (Berl). 191, 461–482. 10.1007/s00213-006-0668-9 17225164

[B71] Salamone J. D. Correa M. Nunes E. J. Randall P. A. Pardo M. (2012). The behavioral pharmacology of effort-related choice behavior: dopamine, adenosine and beyond. J. Exp. Anal. Behav. 97, 125–146. 10.1901/jeab.2012.97-125 22287808 PMC3266736

[B72] Salamone J. D. Yohn S. E. López-Cruz L. San Miguel N. Correa M. (2016). Activational and effort-related aspects of motivation: neural mechanisms and implications for psychopathology. Brain 139, 1325–1347. 10.1093/brain/aww050 27189581 PMC5839596

[B73] Schultz W. Dayan P. Montague P. R. (1997). A neural substrate of prediction and reward. Science 275, 1593–1599. 9054347 10.1126/science.275.5306.1593

[B74] Schultz W. Dickinson A. (2000). Neural coding of prediction errors. Annu. Rev. Neurosci. 23, 473–500. 10.1146/annurev.neuro.23.1.473 10845072

[B75] Schweimer J. Hauber W. (2006). Dopamine D1 receptors in the anterior cingulate cortex regulate effort-based decision making. Learn. Mem. 777–782. 10.1101/lm.409306 17142306 PMC1783632

[B76] Schweimer J. Hauber W. Schweimer J. Hauber W. (2005). Involvement of the rat anterior cingulate cortex in control of instrumental responses guided by reward expectancy. Learn. Mem. 12, 334–342. 10.1101/lm.90605 15930509 PMC1142463

[B77] Subiaul F. Cantlon J. F. Holloway R. L. Terrace H. S. (2004). Cognitive imitation in rhesus macaques. Science 305, 407–410. 10.1126/science.1099136 15256673

[B78] van Reekum R. Stuss D. T. Ostrander L. (2005). Apathy: why care? J. Neuropsychiatry Clin. Neurosci. 17, 7–19. 10.1176/jnp.17.1.7 15746478

[B79] Walsh M. M. Anderson J. R. (2012). Learning from experience: event-related potential correlates of reward processing, neural adaptation, and behavioral choice. Neurosci. Biobehav. Rev. 36, 1870–1884. 10.1016/j.neubiorev.2012.05.008 22683741 PMC3432149

[B80] Walton M. E. Bannerman D. M. Alterescu K. Rushworth M. F. S. (2003). Functional specialization within medial frontal cortex of the anterior cingulate for evaluating effort-related decisions. J. Neurosci. 23, 6475–6479. 12878688 10.1523/JNEUROSCI.23-16-06475.2003 PMC6740644

[B81] Westbrook A. Braver T. (2015). Cognitive effort: A neuroeconomic approach. Cogn. Affect. Behav. Neurosci. 15, 395–415. 10.3758/s13415-015-0334-y 25673005 PMC4445645

[B82] Westbrook A. Braver T. S. (2016). Dopamine does double duty in motivating cognitive effort. Neuron 89, 695–710. 10.1016/j.neuron.2015.12.029 26889810 PMC4759499

[B83] Williams G. V. Goldman-Rakic P. S. (1995). Modulation of memory fields by dopamine D1 receptors in prefrontal cortex. Nature 376, 572–575. 10.1038/376572a0 7637804

[B84] Yoshida K. Saito N. Iriki A. Isoda M. (2011). Representation of others' action by neurons in monkey medial frontal cortex. Curr. Biol. 21, 249–253. 10.1016/j.cub.2011.01.004 21256015

[B85] Yoshida K. Saito N. Iriki A. Isoda M. (2012). Social error monitoring in macaque frontal cortex. Nat. Neurosci. 15, 1307–1312. 10.1038/nn.3180 22864610

[B86] Zahm D. S. (2000). An integrative neuroanatomical perspective on some subcortical substrates of adaptive responding with emphasis on the nucleus accumbens. Neurosci. Biobehav. Rev. 24, 85–105. 10.1016/S0149-7634(99)00065-2 10654664

