# Supplementary material for: Role of Anterior Cingulate Cortex in Instrumental Learning: Blockade of Dopamine D1 Receptors Suppresses Overt but Not Covert Learning
Source: Front Behav Neurosci. 2017 May 15;11:82. doi: 10.3389/fnbeh.2017.00082 (PMC5430040; doi:10.3389/fnbeh.2017.00082)
Supplement: Supplementary file 1 [file Table1.DOCX]

# Supplemental Information

# Supplemental Results

# *Supplemental Tables*

Table S1. Mean values and standard error of the means (SEM) for the dependent variables for the different groups of rats, during the key sessions.
