# Supplementary material for: Role of Anterior Cingulate Cortex in Instrumental Learning: Blockade of Dopamine D1 Receptors Suppresses Overt but Not Covert Learning
Source: Front Behav Neurosci. 2017 May 15;11:82. doi: 10.3389/fnbeh.2017.00082 (PMC5430040; doi:10.3389/fnbeh.2017.00082)
Supplement: Supplementary file 2 [file Table2.DOCX]

# Supplemental Information

# Supplemental Results

# *Supplemental Tables*

Table S2. Effect of treatment: 3-way ANOVA for three key dependent variables: Correct LPs, PCR and latency.

| **Number of LPs** | DF | F | P | η^2^ |
| --- | --- | --- | --- | --- |
| Learning Type | 1.00 | 215.68 | 0.0000 | 0.05 |
| Session | 29.00 | 38.74 | 0.0000 | **0.24** |
| Treatment | 1.00 | 966.48 | 0.0000 | **0.21** |
| Treatment * Session | 29.00 | 38.74 | 0.0000 | **0.24** |
| Treatment * Learning Type | 1.00 | 215.68 | 0.0000 | 0.05 |
| Session * Learning Type | 29.00 | 6.89 | 0.0001 | 0.03 |
| Treatment * Session * Learning Type | 29.00 | 6.89 | 0.0001 | 0.03 |
| **PCR** |  |  |  |  |
| Learning Type | 1.00 | 84.79 | 0.0000 | 0.02 |
| Session | 29.00 | 39.13 | 0.0000 | **0.24** |
| Treatment | 1.00 | 1433.73 | 0.0000 | **0.30** |
| Treatment * Session | 29.00 | 39.13 | 0.0000 | **0.24** |
| Treatment * Learning Type | 1.00 | 84.79 | 0.0000 | 0.02 |
| Session * Learning Type | 29.00 | 4.07 | 0.0000 | 0.03 |
| Treatment * Session * Learning Type | 29.00 | 4.07 | 0.0000 | 0.03 |
| **Latency** |  |  |  |  |
| Learning Type | 1.00 | 0.00 | NS | 0.00 |
| Session | 29.00 | 2.33 | 0.0000 | 0.04 |
| Treatment | 1.00 | 1003.49 | 0.0000 | **0.55** |
| Treatment * Session | 29.00 | 2.75 | 0.0000 | 0.04 |
| Treatment * Learning Type | 1.00 | 0.00 | NS | 0.00 |
| Session * Learning Type | 29.00 | 0.00 | NS | 0.02 |
| Treatment * Session * Learning Type | 29.00 | 0.00 | NS | 0.02  10 |
