# Supplementary figures and images for: Role of Anterior Cingulate Cortex in Instrumental Learning: Blockade of Dopamine D1 Receptors Suppresses Overt but Not Covert Learning
Source: Front Behav Neurosci. 2017 May 15;11:82. doi: 10.3389/fnbeh.2017.00082 (PMC5430040; doi:10.3389/fnbeh.2017.00082)

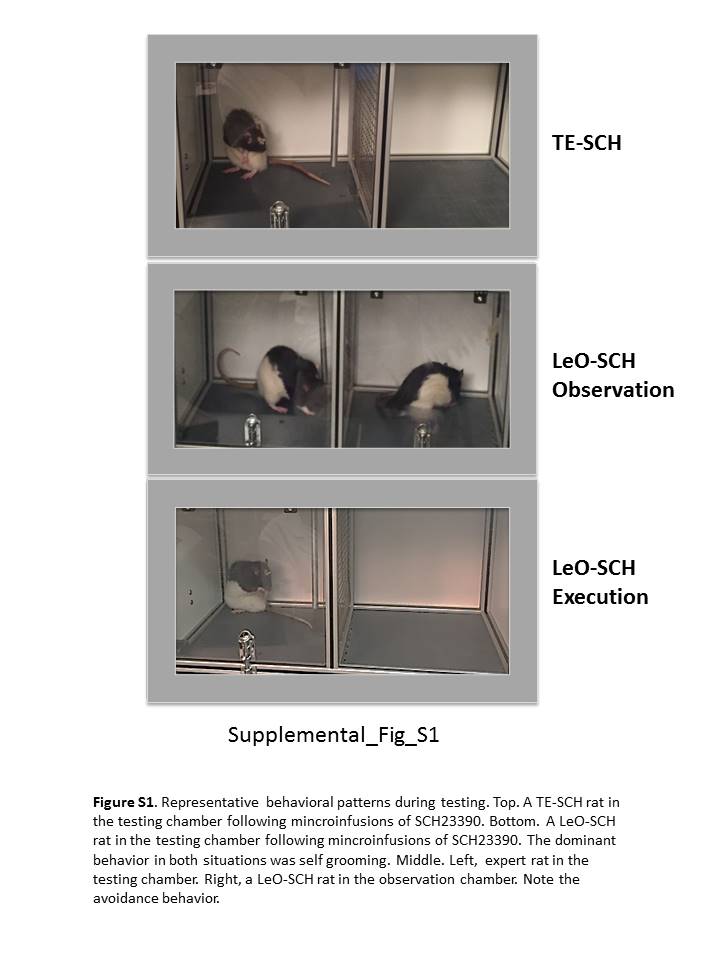

Supplement: Supplementary file 3 [file Image1.JPEG]

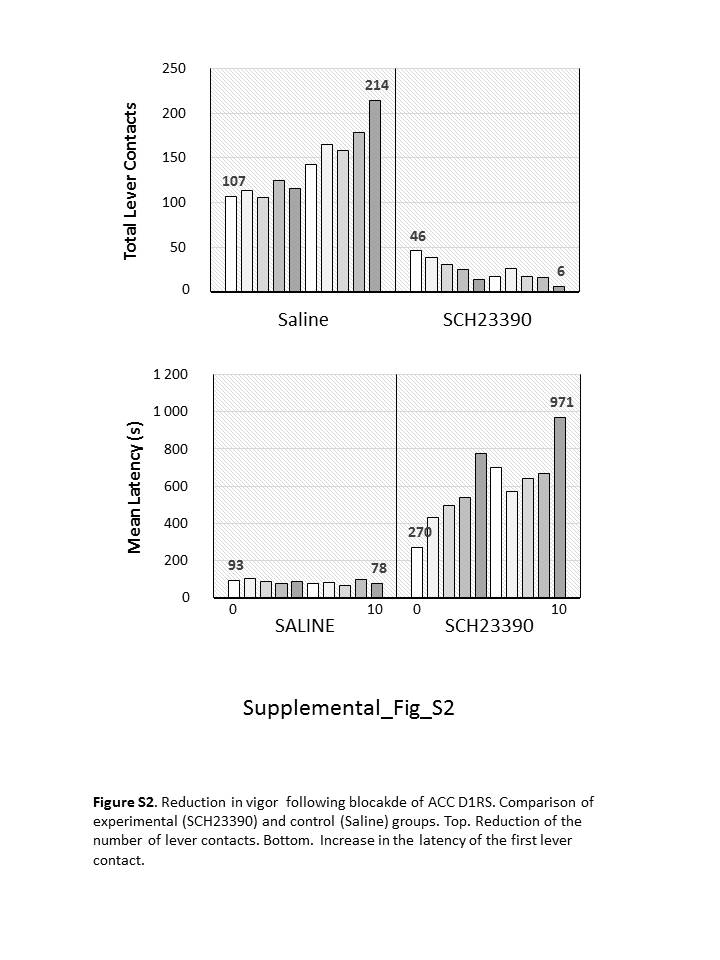

Supplement: Supplementary file 4 [file Image2.JPEG]

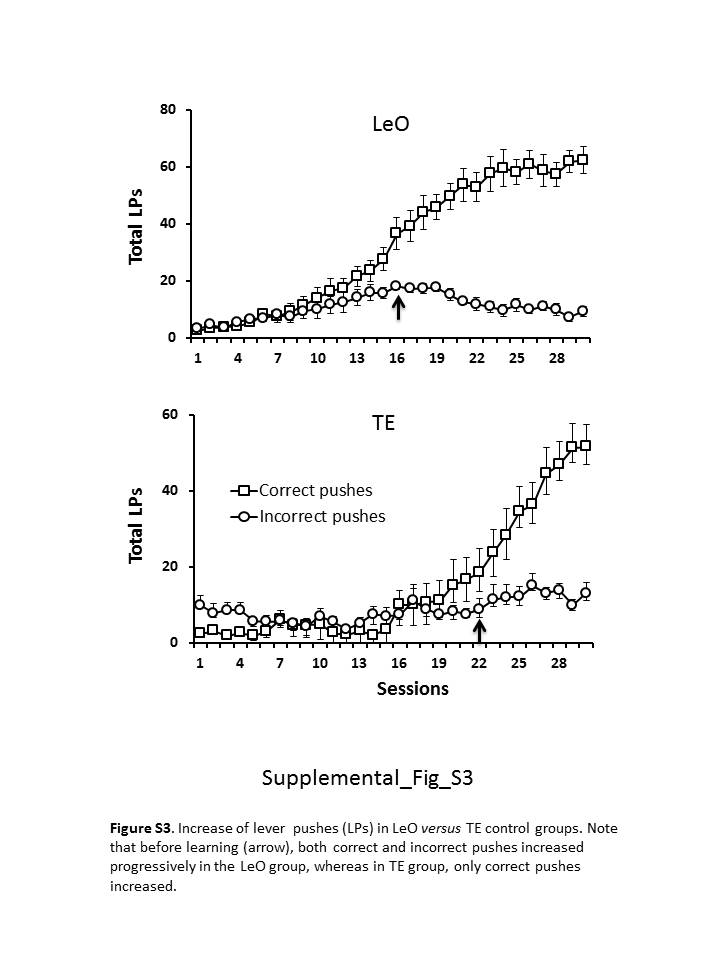

Supplement: Supplementary file 5 [file Image3.JPEG]
